# Evidence of Oocyte Polarity in Bovine; Implications for Intracytoplasmic Sperm Injection and Somatic Cell Nuclear Transfer

**DOI:** 10.22074/cellj.2017.4887

**Published:** 2017-08-19

**Authors:** Seyed Morteza Hosseini, Fariba Moulavi, Nima TanhaieVash, Naser Shams-Esfandabadi, Mohammad Hossein Nasr-Esfahani, Abolfazl Shirazi

**Affiliations:** 1. Research Institute of Animal Embryo Technology, Shahrekord University, Shahrekord, Iran; 2. Department of Reproductive Biotechnology, Reproductive Biomedicine Research Center, Royan Institute for Biotechnology, ACECR, Isfahan, Iran; 3. Reproductive Biotechnology Research Center, Avicenna Research Institute, ACECR, Tehran, Iran

**Keywords:** Oocyte, Polarity, Transcript, Sperm, Bovine

## Abstract

**Objective:**

We recently demonstrated spatial regionalization of maternal transcripts and proteins within unfertilized ovine oocyte. Here, we investigated the likelihood of oocyte polarity for the first time in bovine.

**Materials and Methods:**

In this experimental study, *in vitro* matured bovine oocytes were used for manual bisection [into oocyte halve that were near-to (HNS) and far-from (FS) spindle] or trisection [into MII-spindle (S), the spindle-side half (NS), and the distal half unassociated with the spindle (FS)]. Prepared pools of oocyte substructures were used for comparative quantitative real-time polymerase chain reaction (RT-qPCR). To map the possible preferential sperm entry point (SEP), the spatial relationship between SEP and MII-spindle was measured 5 hours post-fertilization.

**Results:**

The proportional amount of maternal mRNA in S oocyte fragment was estimated to be 6 to 11-fold higher than NS and FS counterparts. The relative abundances of *Nanog, Oct4, Fgf4* and *Tead4* were significantly higher in HNS oocyte fragment compared t0 FS. The relative abundances of *Ctnb, Carm1, Rex1, Sox2* and *Cdx2* were comparable between HNS and NS oocyte fragments. FS oocyte fragment possessed significantly higher transcripts of *Gata4* compared to HNS. The distribution of certain transcripts related to pluripotency and lineage commitment were different depending upon the region of the oocyte; either enriched at S (*Tead4, Nanog, Ctnb* and *Sox2*), NS (*Oct4*), or FS (*Gata6*). The SEP in almost (90%) fertilized oocytes was located in MII-hemisphere.

**Conclusion:**

The observation of spatial restriction of mRNAs and SEP within MII-oocyte may indicate that the principal forces of oocyte polarity are evolutionary conserved. This may in turn highlight the need for refinements in the methodology of intracytoplasmic sperm injection (where a sperm is injected far from the MII-spindle) and somatic cell nuclear transfer (where a major amount of regulative mRNAs that are associated with MIIspindle is removed during enucleation).

## Introduction

Polarity and asymmetry are characteristic features of most cells in all organisms throughout development (1). Within the oocytes of lower vertebrates and invertebrates, protein domains and their concentration gradients are polarized (2-4). However, whether the same form of oocyte polarity also exists in mammals is currently under hot debate (5-8). The conceptual controversy of mammalian oocyte polarity has significant implications for not only theory and research, but also assisted reproduction techniques (ART). For many years, it has been recommended that during intracytoplasmic sperm injection (ICSI), sperm should be injected far from the first polar body (9). Somatic cell nuclear transfer (SCNT) is carried out by removing a few or half of the oocyte cytoplasm near to MII-spindle during oocyte enucleation (10). These techniques have been established based on a long-lasting concept that in mammals, the oocyte is nonpolar and the blastomeres of early embryo are equal in their competence. Even though, if further studies could support the existence of cytoplasmic "polarity" of oocyte, refinements may need to be considered in these manipulative and diagnostic ARTs (11).

Investigation of oocyte polarity is poorly understood in mammalian species other than mice. We recently developed a handmade method for microsurgical trisection of unfertilized MIIoocytes into cortical cytoplasm around spindle (S), and cytoplast hemispheres that were located either near (NS) or far (FS)-to-spindle. Using a series of manipulative, developmental and molecular studies, we showed for the first time that maternal transcripts and proteins are polarized in MII oocytes of ovine, as a large mammalian species. The bovine is an ideal species for production of agriculture and transgenic production of pharmaceutical proteins. Accordingly, a clear understanding of oocyte and embryo polarity has the pivotal importance for monozygotic twining, derivation and establishment of embryonic stem cell, and prediction of the developmental competence of the corresponding sister blastomere (11). Therefore, this study was carried out for the first time in bovine to investigate two important features of oocyte polarity; i. The spatial distribution of maternal mRNAs and ii. The possible preferential topological point of sperm penetration.

## Materials and Methods

### Oocyte preparation

In this experimental study, the procedure of oocyte *in vitro* maturation (IVM) was as described previously (12). In brief, antral follicles (2-6 mm diameter) of abattoir-derived ovaries were aspirated to obtain cumulusoocyte complexes (COCs). Selected COCs with homogenous cytoplasm and more than three layers of surrounding cumulus cells were washed three times in hepes-buffered tissue culture medium 199 (HTCM199, Gibco, USA)+10% sheep serum (SS) followed by three further washing in maturation medium [TCM199 (Gibco, USA) containing 2.5 mM Na-pyruvate (Sigma, USA), 1 mM L-glutamine (Gibco, USA), 100 IU/ml penicillin (Sigma, USA), 100 μg/ml streptomycin (Sigma, USA), 10% fetal calf serum (FCS, Gibco, USA), 10 μg/ml ovine follicle stimulating hormone (FSH, Sigma, USA), 10 μg/ml ovine luteinizing hormone (LH, Sigma, USA), 1 μg/ml estradiol- 17ß (Sigma, USA), and 0.1 mM cysteamine (Sigma, USA)]. Oocytes were then cultured for 20-22 hours in groups of 20-25 in 100 μl droplets of maturation medium covered with mineral oil at 38.5ºC, 5% CO_2_ humidified air. Matured COCs were then used for embryo development and presumptive zygotes were cultured in groups of 6-8 in 20 μl droplets of a modified formulation of synthetic oviduct fluid described (mSOF) at 39ºC, 6% CO_2_, 5% O_2_ and humidified air for 168 hours.

### Spatial distribution of maternal mRNAs in bovine oocytes

To understand the spatial distribution of transcripts within MII-oocytes, a manual method of oocyte trisection was used as described previously (13). In brief, MII-oocytes (n=290) were first treated with pronase (0.05% in HTCM199) to remove the zona. These zona-free oocytes were then treated with 0.4 mM demoecolcine (Sigma, USA) for 0.5 hours to induce partial extrusion of MII-spindle and associated chromosomes as a clearly visible cytoplasmic protrusion. Treated oocytes were placed in groups of 5-to-10 in the droplets of enucleation medium that prepared in the 35mm culture dishes (Greiner, Cellstar, 27160) under mineral oil. The diameter of zona-free oocytes were approximately 100-120 μm. Pasteur glass pipette were pulled on the flame of a burner in two steps to produce two types of manipulation devices with inner diameter: i. Approximately half the oocyte diameter (≈50-60 μm), and ii. Slightly larger than the cytoplasmic protrusion (20-30 μm). "Only pipettes with completely smooth tip orifice were selected" (13). To provide a very gentle and controlled suction on the tip, the pipette was backfilled with a large column (1-1.5 ml) of mineral oil to neutralize capillary action.

**Fig.1:**
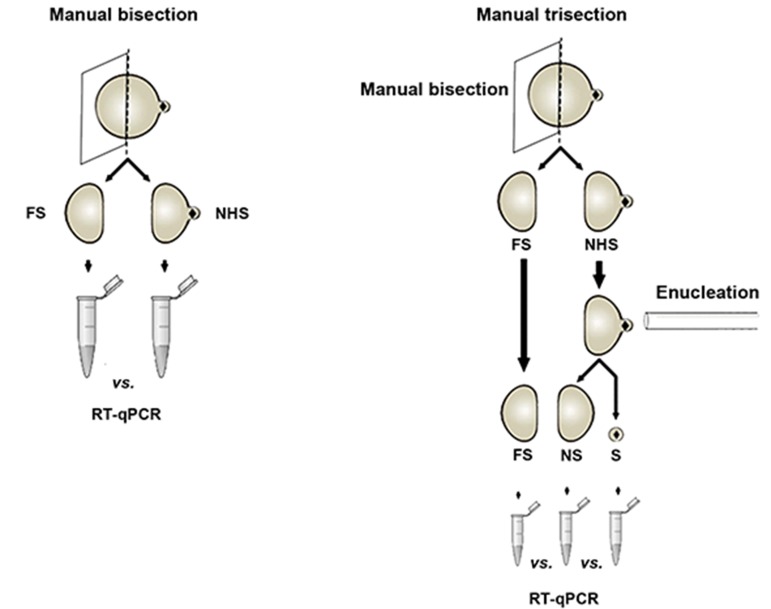
The schematic representation of methods of manual oocyte bisection (left) and trisection (right). FS; Far from spindle, HNS; Halve near to spindle, NS; Near to spindle, S; MII-spindle, and RT-qPCR; Quantitative real-time polymerase chain reaction.

For bisection, the oocytes were gently rolled using pipette tip under a stereomicroscope until the cytoplasmic protrusion was adjusted at 3 O’clock. Then the tip of the pipette was put close to the oocyte and under controlled suction, the oocyte half near to the MII-spindle was sucked into the pipette. Meanwhile, the pipette moved out from the droplet of medium to the mineral oil. The result was bisection of oocyte to two halves with reference to MIIchromosome: the half near to MII-chromosome (HNS) and the half far from (FS) spindle (Fig .1Left). The HNS halves were used for manual enucleation using type II of manipulation device. In brief, HNS halves were rotated using the tip of the device until the cytoplasmic protrusion was fitted on the cytoplasmic protrusion. As soon as the cytoplasmic protrusion was entered into the tip, the pipette was moved out from the droplet of medium to the mineral oil. The result was separation of the HNS halves to two parts: i. MII-chromosomes enclosed in a scant cytoplasm (S), ii. Majority of HNS part without MII-chromosome (NS) (Fig .1Right). To confirm successful bisection and spindle removal, oocytes were stained with Hoechst 33342 (5 μg/ml, 5 minutes) before microsurgery. The trisected oocytes were then visualized by brief UV-exposure. The pools of HNS, NS, FS and S -cytoplasmic fragments in minimum extraneous media were transferred into RLT buffer to be kept in frozen until RNA extraction and quantitative real-time polymerase chain reaction (RT-qPCR).

### Topological assessment of sperm entry point in the bovine eggs

**Fig.2:**
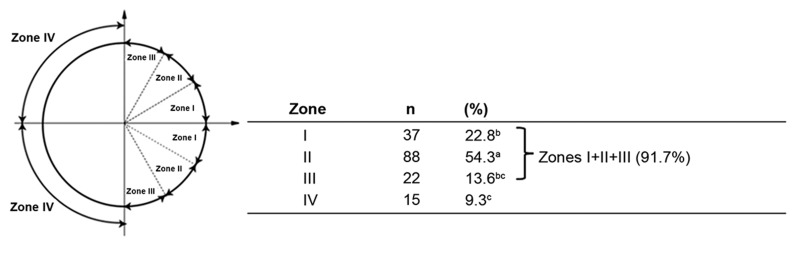
The topological distribution of sperm entry position in *in vitro* fertilized bovine oocytes. ^a-c^; Values with common letter are not significantly different (P<0.05).

To investigate whether sperm entry point (SEP) is random or preferential in bovine, oocytes (n=311) were submitted to *in vitro* fertilization (IVF) as described previously (10). In brief, frozen-thawed and washed sperm from a single Holstein sire of proven *in vitro* fertility were used for fertilization after capacitation by the swim-up procedure. Spermatozoa (1×10^6^/ ml sperm) and matured COCs (40-45 COCs/200 μl) were co-incubated in modified fertilization- Tyrode’s albumin lactate pyruvate (TALP, handmade) medium containing 0.01 mM heparin (Sigma, USA), 0.2 mM penicillamine (Sigma, USA) and 0.1 mM hypotaurine (Sigma, USA) for 18 hours at 39.5˚C, 6% CO_2_ in humidified air. Fertilized oocytes at 4-5 hours post fertilization (hpf) were fixed, stained with Hoechst-33342 and visualized with a fluorescence-assisted micromanipulator microscope (Olympus, Japan). Schurmann et al. (14) demonstrated 4-6 hpf as the optimum timepoint of maximum early fertilization. To map the SEP, eggs were rotated under constant UVlight until MII-spindle (the approved reference point of SEP) was positioned to 3 O’clock. Then, the spatial relationship between SEP and MII-spindle was measured as described by Motosugi et al. (Fig .2) (15). In this scheme, zones I and IV are the closest and the furthest from the MII-spindle, respectively.

### Quantitative real-time polymerase chain reaction

The transcript abundances of 10 genes that are related to pluripotency and lineage commitment (Table 1) were compared between NS and FS oocyte halves. The procedure of RT-qPCR was as described previously (16). In brief, total RNA was extracted suing RNeasy Micro kit (Qiagen, Mississauga, ON, Canada) followed by the treatment with DNase-I (Ambion, Streetsville, ON, Canada) according to the manufacturer’s protocol. The RNA quality and quantity was determined using the WPABiowave spectrophotometer (Cambridge, UK). For reverse transcription, 10 μl of total RNA was used in a final volume of 20 ml reaction containing 1 μl of Random Hexamer, 4 μl RT buffer (10X), 2 μl of dNTP, 1 μl of RNase inhibitor (20 IU), and 1 μl of reverse transcriptase (Fermentas, Glen Burnie, Ontario, Canada). Reverse transcription was carried out at 25˚C for 10 minutes, 42˚C for 1 hour and 70˚C for 10 minutes. The master mix was prepared using 1 μl of cDNA (50 ng), 5 μl of the SYBR Green/0.2 μl ROX qPCR Master Mix (2X, Fermentas, Germany) and 1 μl of forward and reverse primers (5 pM) adjusted to a total volume of 10 μl using water nucleasefree. Three technical replicates of RT-qPCR were conducted for each primer. CT samples of each target gene were normalized to the CT of *Gapdh* (because of its stable expression between groups) and represented as 2^-ΔΔCT^ (17). The primer sequences, annealing temperatures and the size of amplified products are shown in Table 1.

### Statistical analysis

All experiments were replicated at least three times. Before any statistical analysis, the normality of data was evaluated. Percentages data were transformed by ArcSin and analyzed by one-way ANOVA model of SPSS version 17 (SPSS, Science, Chicago, IL, USA). Differences were considered as significant at P<0.05.

## Results

### Spatial distribution of maternal mRNAs in bovine oocytes

The mean mRNAs content of S fragments was significantly lower than NS and FS fragments (4.1 vs. 8.5 vs. 11.0 ng/μl, respectively). Even though, mean mRNAs content of NS was closely similar to FS parts. The RT-qPCR comparison of transcripts between HNS and FS revealed differential distribution of some transcripts assessed (Figes. 1, 3). Particularly, the relative abundances of *Nanog, Oct4, Fgf4* and *Tead4* were significantly higher in HNS rather than FS fragments. The relative abundances of *Ctnb, Carm1, Rex1, Sox2* and *Cdx2* were comparable between HNS and NS fragments. The FS possessed significantly higher transcripts of Gata4 compared to HNS (Table 2).

**Table 1:** Specific primers used in this study


Gene	Primer sequence (5ˊ-3ˊ)	Melting temprature (˚C)	Length

*Oct4*	F: GGAAAGGTGTTCAGCCA	58	110
	R: ATTCTCGTTGTTGTCAGC		
*Nanog*	F: ATTCTTCCACAAGCCCT	60	125
	R: CATTGAGCACACACAGC		
*Sox2*	F: ATGGGCTCGGTGGTGA	52	182
	R: CTCTGGTAGTGCTGGGA		
*Tead4*	F: CTGACAAGAGTGTGGAGAAG	62	114
	R: CTACCCATAGGATACAAAGC		
*Gata4*	F: TCCCCTTCGGGCTCAGTGC	63	108
	R: GTTGCCAGGTAGCGAGTTTGC		
*Carm1*	F: CTCCAAGTCCAGTAACCT	60	120
	R: CCGCTGCTGAGATTATAG		
*Cdx2*	F: CCCCAAGTGAAAACCAG	56	107
	R: TGAGAGCCCCAGTGTG		
*Fgf4*	F: GTGATGTCTGGTCCTTCG	55	105
	R: GAAGGTGGGTCTCTGTGA		
*Rex1*	F: GCAGCGAGCCCTACACAC	59	117
	R: ACAACAGCGTCATCGTCCG		


The RT-qPCR comparison of transcripts between S, NS and FS also revealed differential distribution of some transcripts assessed (Figes. 2, 4). Accordingly, the S fragment possessed significantly higher transcripts of *Tead4* and *Nanog* compared to both NS and FS counterparts and of *Ctnb* and *Sox2* compared to FS counterpart. The NS fragment possessed significantly higher amount of *Oct4* compared to both S and FS counterparts. The FS fragment possessed significantly higher amount of Gata6 compared to both S and NS counterparts. The relative abundances of *Fgf4, Carm1, Rex1* and *Cdx2* were comparable between S, NS, and FS parts.

### Topological assessment of sperm entry point in bovine eggs

Figure 2 represents the topological assessment of SEP with reference to MII-chromosomes. As shown, SEP in almost all (91.7%) fertilized oocytes was located in MII-hemisphere (zones I-III) compared to non-MII-hemisphere (zone IV: 9.3%). Within MII-hemisphere 22.8, 54.3, 13.6% of SEPs were located in zones I, II, and III, respectively.

**Table 2:** The relevant information on developmental roles of genes assessed in this study


Gene	Relevant information	Reference

*Cdx2*	Involved in TE differentiation. Also involved in the transcriptional regulation of multiple genes expressed in the intestinal epithelium. Important in broad range of functions from early differentiation to maintenance of the intestinal epithelial lining of both the small and large intestine.	(18)
*Carm1*	A gene in the family of protein arginine methyltransferase (PRMT). The encoded enzyme may act in association with other proteins or within multi-protein complexes. CARM1 directs embryonic cells toward inner cell mass formation through elevation of expression of key pluripotency genes.	(19)
*Tead4*	This gene product is a member of the transcriptional enhancer factor (TEF) family of transcription factors, which contain the TEA/ATTS DNA-binding domain. TEAD4 is considered an upstream regulator of cell linage commitment in early embryo toward trophectoderm.	(20)
*Gata6*	This gene encodes a member of the GATA family of zinc-finger transcription factors. Members of this family recognize the GATA motif which is present in the promoters of many genes. GATA6 interaction with NANOG is considered a main regulator of epiblast and hypoblast formation in inner cell mass cells of the mature blastocyst.	(21)
*Ctnb*	Catenin beta 1, also called beta-catenin (or β-catenin), is a dual function protein, regulating the coordination of cell–cell adhesion and gene transcription. CTNB is the nuclear effector of WNT signaling pathway.	(22)
*Oct4*	Transcription factor that binds to the octamer motif (5’-ATTTGCAT-3’). Forms a trimeric complex with SOX2 on DNA and controls the expression of a number of genes involved in embryonic development. Critical for early embryogenesis and for embryonic stem cell pluripotency.	(23)
*Rex1*	Involved in the reprogramming of X-chromosome inactivation during the acquisition of pluripotency. Required for efficient elongation of TSIX, a non-coding RNA antisense to XIST. Binds DXPas34 enhancer within the TSIX promoter. Involved in ES cell self-renewal.	(23)
*Sox2*	SRY (sex determining region Y)-box 2, also known as SOX2, is a transcription factor that is essential for maintaining self-renewal, or pluripotency, of undifferentiated embryonic stem cells. and have been shown to play key roles in many stages of mammalian development.	(23)
*Nanog*	Transcription regulator involved in inner cell mass and embryonic stem (ES) cells proliferation and self-renewal. Imposes pluripotency on ES cells and prevents their differentiation towards extra-embryonic endoderm and trophectoderm lineages.	(23)
*Tead4*	Transcription factor GATA-4 is a protein that in humans is encoded by the Gata4 gene. This protein is thought to regulate genes involved in embryogenesis and in myocardial differentiation and function.	(24)
*Gapdh*	A highly conserved protein that is involved in various types of cell motility and is ubiquitously expressed in all eukaryotic cells.	(25)


**Fig.3:**
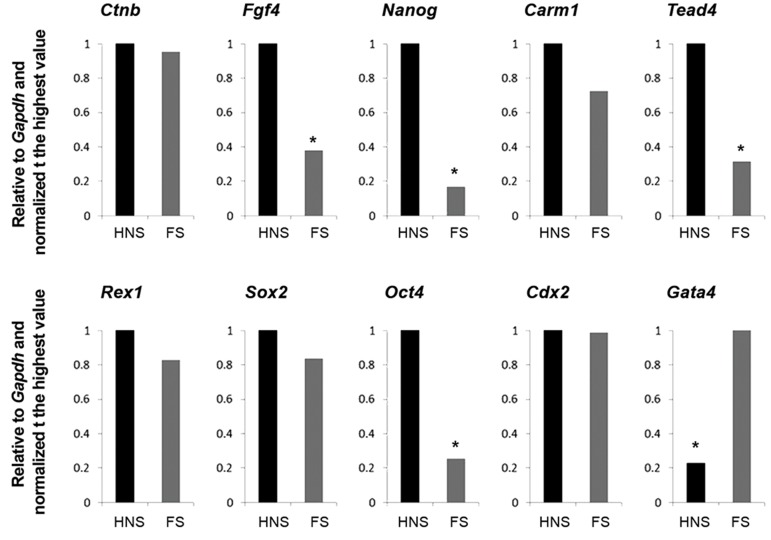
RT-qPCR analysis of relative abundances of transcripts between demi-oocytes prepared in relation to the MII-spindle reference point. RT-qPCR; Quantitative real-time polymerase chain reaction, FS; Far from spindle, HNS; Halve near to spindle, and *; Significant difference between HNS and FS groups.

**Fig.4:**
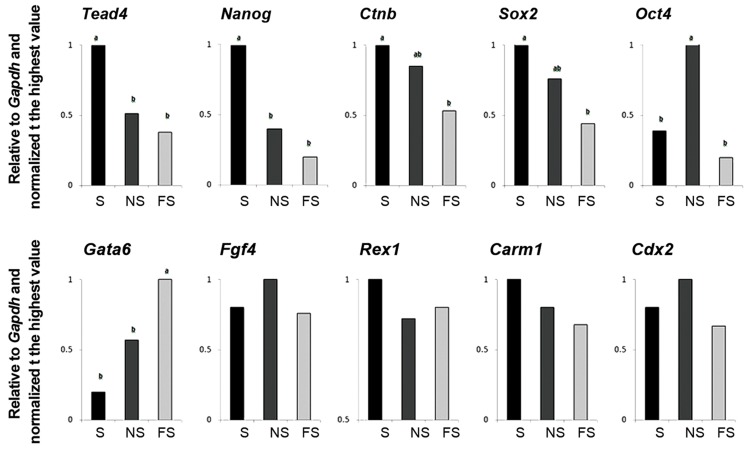
RT-qPCR analysis of relative abundances of transcripts between oocyte fragments prepared in relation to the MII-spindle reference point. Values with different letters indicate significant difference within groups. RT-qPCR; Quantitative real-time polymerase chain reaction, NS; Near to spindle, FS; Far from spindle, and S; MII-spindle.

## Discussion

To best of our knowledge, this is the first study that provides evidence of transcriptional regionalization of some developmentally important genes within MII bovine oocytes. We also demonstrated the first topological evidence of preferential sperm entry during IVF of bovine MIIoocytes. These results may open the debate again as whether mammalian and human oocytes are polar which may in turn raise important questions about the subsequent effects of oocyte polarity on ART methodology. It is particularly important that the MII-chromosomes had a central role in topological patterns observed in the distribution of transcripts and SEP. In this sense, the oocyte hemisphere that was nearer to the MII-chromosomes possessed higher abundances of some developmentally important transcripts rather than far than MIIchromosomes hemisphere. Moreover, almost sperm entry points were detected in the MIIhemisphere. This may suggest that the oocyte hemisphere near to MII-chromosomes is enriched for those maternal instructions that are required for: i. Attraction of fertilizing sperm, ii. Paternal chromosome decondensation/reprogramming, and iii. Early embryonic divisions until zygote genome activation (ZGA).

The asymmetric localization of maternal transcripts is an essential polarity determinant, directing cis-regulation of zygotic genes in metazoan (18, 26, 27). In vertebrates, the polarity of oocyte is perhaps best-documented in amphibians, where the strict cytoplasmic regionalization of maternal molecules and clues along the animalvegetal axis has emerged as a fundamental mechanism of embryonic development and lineage commitment (18). In our study, the mean mRNAs content of the scant cytoplasm associated with MII-chromosome was in the range of half and one-third associated amounts of NS and FS counterparts. Even though, considering the fact that S constitutes only 2-3% of total oocyte volume, the proportional amount of mRNAs that is associated with the MII-spindle could be estimated in a range of 6 to 11 -fold compared to NS and FS counterparts. This may suggest a critical role of MII-chromosome topology in the regulation of early event of fertilization *in vitro* that is likely to affect subsequent events of embryo development. Although conservation of a similar form of oocyte polarity in mammals is assumed, recent reports are conflicting and there exist evidence both for (26, 28-32) and against (33, 34) the idea of oocyte polarity in mouse. Therefore, our observation of spatial restriction of transcripts and SEP in bovine oocytemay suppose the existence of a similar form of polarity in mammals. In agreement with our results, (32) demonstrated evidence of transcriptional regionalization within unfertilized mouse oocytes. They showed marked differences in the transcriptome profiles of MII-spindle and first polar body (of unfertilized oocyte) and the second polar body (of fertilized oocytes) compared to the oocyte cytoplasm. Although the results of this study may provide support for the first group of researchers, it is peculiarly important to determine whether this oocyte polarity is temporary (due to asymmetric localization of molecules) or is of a permanent, irreversible and irreplaceable nature in all cells of the same type (as observed in amphibian oocytes) (11, 35).

The preferential sperm entry into animal pole point is well-documented in amphibian oocytes. This phenomenon has also been demonstrated in mouse oocyte by two other studies in mice (6, 15). Even though, while the first group (6) demonstrated that sperm preferentially enter into the "vegetal" area of mice oocyte, the second group believes in apposite site of SEP (15). Here, we observed that majority (90%) of bovine oocytes are fertilized through the MII-hemisphere. Piotrowska and Zernicka-Goetz (6) provided evidence that SEP has a determining role in spatial patterning of the mouse early embryos. In contrast, Hiiragi and Solter (33) demonstrated that it is the topological relationship between the parental pronuclei, rather than the SEP, that determines the cleavage plane of mouse embryo. While the reason of this controversy is not understood, further studies are required to elucidate whether our observation of preferential SEP in bovine has any bearing effect on the contribution of the first two blastomeres to the blastocyst development. From a practical point of view, our observation of spatial restriction of mRNAs and SEP may highlight the need for further studies, even refinements, in the methodology of ICSI and SCNT in bovine, at least. Perhaps for example, if sperm might naturally enter through the MIIhemisphere which is enriched for maternal mRNAs, forced injection of sperm far from MII-spindle could affect the developmental competence of ICSI-embryos. In the same way, these results may be relevant to low developmental competence of SCNT embryos where a great source of regulative maternal mRNAs and perhaps proteins are removed during oocyte enucleation. Further studies are needed to understand if this hypothetical oocyte polarity in transcripts has important during ICSI and SCNT in bovine (11). 

## Conclusion

To the best of our knowledge, this is the first study that provides evidence of spatial restriction of developmentally important transcripts and sperm entry point in the bovine MII-oocytes. These results although are preliminary for the controversial issue of oocyte polarity in mammals, may highlight the need for further studies, even refinements, in the methodology of ICSI and SCNT in bovine, at least. Even though, future studies should be focused on the possible relationship between SEP, zona pelucida, first cleavage plane of embryos, and the contribution of the first blastomeres to the embryonal axis formation in bovine which in turn would illuminate the way for *in vitro* manipulation for *in vitro* capturing pluripotent cells in this valuable farm species.

## References

[1] Plancha CE, Sanfins A, Rodrigues P, Albertini D (2005). Cell polarity during folliculogenesis and oogenesis. Reprod Biomed Online.

[2] Marlow FL (2010). Maternal control of development in vertebrates: my mother made me do it! Oocyte polarity and the embryonic axes: the balbiani body, an ancient oocyte asymmetry.

[3] Moen TL, Namenwirth M (1977). The distribution of soluble proteins along the animal-vegetal axis of frog eggs. Dev Biol.

[4] Jäckle H, Eagleson GW (1980). Spatial distribution of abundant proteins in oocytes and fertilized eggs of the Mexican axolotl (Ambystoma mexicanum).Devi Biol.

[5] Weber RJ, Pedersen RA, Wianny F, Evans MJ, Zernicka- Goetz M (1999). Polarity of the mouse embryo is anticipated before implantation. Development.

[6] Piotrowska K, Zernicka-Goetz M (2001). Role for sperm in spatial patterning of the early mouse embryo. Nature.

[7] Motosugi N, Bauer T, Polanski Z, Solter D, Hiiragi T (2005). Polarity of the mouse embryo is established at blastocyst and is not prepatterned. Genes Dev.

[8] Hiiragi T, Alarcon VB, Fujimori T, Louvet-Vallee S, Maleszewski M, Marikawa Y (2006). Where do we stand now?. Mouse early embryo patterning meeting in Freiburg, Germany (2005). Int J Dev Biol.

[9] Eichenlaub-Ritter U, Shen Y, Tinneberg HR (2002). Manipulation of the oocyte: possible damage to the spindle apparatus. Reprod Biomed Online.

[10] Moulavi F, Hosseini SM, Hajian M, Forouzanfar M, Abedi P, Ostadhosseini S (2013). Nuclear transfer technique affects mRNA abundance, developmental competence and cell fate of the reconstituted sheep oocytes. Reproduction.

[11] Fulka J Jr, Kárníková L, Moor RM (1998). Oocyte polarity: ICSI, cloning and related techniques. Hum Reprod.

[12] Hosseini SM, Moulavi F, Hajian M, Abedi P, Forouzanfar M, Ostad-Hosseini S (2008). Highly efficient in vitro production of bovine blastocyst in cell-free sequential synthetic oviductal fluid vs.TCM 199 Vero cell co-culture system. Int J Fertil Steril.

[13] Hosseini SM, Hajian M, Moulavi F, Asgari V, Forouzanfar M, Nasr-Esfahani MH (2013). Cloned sheep blastocysts derived from oocytes enucleated manually using a pulled pasteur pipette. Cell Reprogram.

[14] Schurmann A, Wells DN, Oback B (2006). Early zygotes are suitable recipients for bovine somatic nuclear transfer and result in cloned offspring. Reproduction.

[15] Motosugi N, Dietrich JE, Polanski Z, Solter D, Hiiragi T (2006). Space asymmetry directs preferential sperm entry in the absence of polarity in the mouse oocyte. PLoS Biol.

[16] Hosseini SM, Asgari V, Ostadhosseini S, Hajian M, Ghanaei HR, Nasr-Esfahani MH (2015). Developmental competence of ovine oocytes after vitrification: differential effects of vitrification steps, embryo production methods, and parental origin of pronuclei. Theriogenology.

[17] Schmittgen TD, Livak KJ (2008). Analyzing real-time PCR data by the comparative C(T) method. Nat Protoc.

[18] Nüsslein-Volhard C (1991). Determination of the embryonic axes of Drosophila. Dev Suppl.

[19] Wu Q, Bruce AW, Jedrusik A, Ellis PD, Andrews RM, Langford CF (2009). CARM1 is required in embryonic stem cells to maintain pluripotency and resist differentiation. Stem Cells.

[20] Nishioka N, Yamamoto S, Kiyonari H, Sato H, Sawada A, Ota M (2008). Tead4 is required for specification of trophectoderm in pre-implantation mouse embryos. Mech Dev.

[21] Chazaud C, Yamanaka Y, Pawson T, Rossant J (2006). Early lineage segregation between epiblast and primitive endoderm in mouse blastocysts through the Grb2-MAPK pathway. Dev Cell.

[22] MacDonald BT, Tamai K, He X (2009). Wnt/beta-catenin signaling: components, mechanisms, and diseases. Dev Cell.

[23] Kimber SJ, Sneddon SF, Bloor DJ, El-Bareg AM, Hawkhead JA, Metcalfe AD (2008). Expression of genes involved in early cell fate decisions in human embryos and their regulation by growth factors. Reproduction.

[24] Jedrusik A, Cox A, Wicher KB, Glover DM, Zernicka- Goetz M (2015). Maternal-zygotic knockout reveals a critical role of Cdx2 in the morula to blastocyst transition. Dev Biol.

[25] Zhu J, He F, Hu S, Yu J (2008). On the nature of human housekeeping genes. Trends Genet.

[26] Herr JC, Chertihin O, Digilio L, Jha KN, Vemuganti S, Flickinger CJ (2008). Distribution of RNA binding protein MOEP19 in the oocyte cortex and early embryo indicates pre-patterning related to blastomere polarity and trophectoderm specification. Dev Biol.

[27] Macara IG, Mili S (2008). Polarity and differential inheritanceuniversal attributes of life?. Cell.

[28] Johnson MH, Eager D, Muggleton-Harris A, Grave HM (1975). Mosaicism in organisation of concanavalin A receptors on surface membrane of mouse egg. Nature.

[29] Edwards RG, Beard HK (1997). Oocyte polarity and cell determination in early mammalian embryos. Mol Hum Reprod.

[30] Duncan FE, Moss SB, Schultz RM, Williams CJ (2005). PAR-3 defines a central subdomain of the cortical actin cap in mouse eggs. Dev Biol.

[31] Antczak M, Van Blerkom J (1997). Oocyte influences on early development: the regulatory proteins leptin and STAT3 are polarized in mouse and human oocytes and differentially distributed within the cells of the preimplantation stage embryo. Mol Hum Reprod.

[32] VerMilyea MD, Maneck M, Yoshida N, Blochberger I, Suzuki E, Suzuki T (2011). Transcriptome asymmetry within mouse zygotes but not between early embryonic sister blastomeres. EMBO J.

[33] Hiiragi T, Solter D (2004). First cleavage plane of the mouse egg is not predetermined but defined by the topology of the two apposing pronuclei. Nature.

[34] Kurotaki Y, Hatta K, Nakao K, Nabeshima Y, Fujimori T (2007). Blastocyst axis is specified independently of early cell lineage but aligns with the ZP shape. Science.

[35] Kloc M, Ghobrial RM, Borsuk E, Kubiak JZ (2012). Polarity an asymmetry during mouse oogenesism and oocyte maturation. Results Probl Cell Differ.

